# Zinc oxide nanoparticles improve lettuce (*Lactuca sativa L.*) plant tolerance to cadmium by stimulating antioxidant defense, enhancing lignin content and reducing the metal accumulation and translocation

**DOI:** 10.3389/fpls.2022.1015745

**Published:** 2022-10-27

**Authors:** Feng Gao, Xiaodan Zhang, Jing Zhang, Jing Li, Tianhang Niu, Chaonan Tang, Cheng Wang, Jianming Xie

**Affiliations:** ^1^ College of Horticulture, Gansu Agricultural University, Lanzhou, China; ^2^ Institute of Vegetables, Gansu Academy of Agricultural Sciences, Lanzhou, China

**Keywords:** cadmium, nanoparticles, lettuce, antioxidants, stress

## Abstract

Cadmium (Cd) contamination is a serious global concern that warrants constant attention. Therefore, a hydroponic study was conducted to evaluate the effect of different concentrations (0, 1, 2.5, 5, 10, 15 mg/l) of zinc oxide nanoparticles (ZnONPs) on the Cd content in lettuce (*Lactuca sativa L.*) under Cd stress conditions. The results showed that Cd stress triggered a decrease in plant biomass, an increase in relative electrolyte conductivity (REC), a decrease in root activity, accumulation of reactive oxygen species (ROS) accumulation, and nutrient imbalance. The application of ZnONPs reduced the toxicity symptoms of lettuce seedlings under Cd stress, with the most pronounced effect being observed 2.5 mg/l. ZnONPs promoted the growth of lettuce under Cd stress, mainly in terms of increase in biomass, chlorophyll content, antioxidant enzyme activity, and proline content, as well as reduction in Cd content, malondialdehyde, and reactive oxygen species (ROS) in plant tissues. ZnONPs also enhanced the uptake of ions associated with photosynthesis, such as iron, manganese, magnesium, and zinc. In addition, ZnONPs increase the amount of lignin in the roots, which blocks or reduces the entry of Cd into plant tissues.

## Introduction

In recent years, Cd contamination in agricultural fields has been escalated by anthropological activities such as mining, irrigation with industrial wastewater, and the application of phosphate fertilizers, herbicides, and pesticides (Seleiman et al., 2020b; Schlutow et al., 2021). Cd is a toxic, non-essential metal that is highly water-soluble and mobile. This eases the uptake of Cd by the roots to take up and send Cd to the leaves through the xylem (Saidi et al., 2014). Trace concentrations of Cd can induce metal toxicity symptoms in plants (Sary Hassan and Ibrahim Ali, 2019). Cadmium complicates the absorption of essential nutrients by plants and accumulates considerably disease in plants (Usman et al., 2019). Eventually, the uptake and transfer of Cd by plants can lead to food chain contamination and health problems, such as anemia, nephrotoxicity and itai-itai Disease (Salem et al., 2018). Therefore, Cd is considered to be one of the most toxic and prevalent heavy metals (Ismael et al., 2019). Cadmium poisoning induces some morphological, physiological, biochemical, and ultrastructural changes in plants (Khan et al., 2021). It causes the causing retarded growth of plant shoots and roots, chlorosis of leaves, and browning of root ends (Zhang et al., 2015). Cd induces the breakdown of proteins and lipids, damages mitochondria, chloroplasts, and cell membrane ultrastructure (Ali et al., 2013b; Howladar et al., 2018), leading to a reduction in chlorophyll content and triggers higher levels of reactive oxygen species (ROS). In addition, it causes an imbalance of nutrient elements (Erdal and Turk, 2016; Santos et al., 2018).Plants mitigate Cd toxicity by modulating their antioxidant mechanisms, such as biomolecules and antioxidant enzymes (Laxa et al., 2019). These stress-related biomolecules and antioxidant enzymes can increase the tolerance of plants to Cd stress. For example, thiols and their metabolites can fix Cd in vesicles to limit uptake (Yan et al., 2020); lignin protects protoplasts by immobilizing Cd into the root cell wall (Parrotta et al., 2015). Peroxidase (POD), involved in lignin synthesis, can help plants to scavenge excess H_2_O_2_ and reduce damage caused by oxidative stress (Jalloh et al., 2009). Several studies have shown that plant growth regulators, elemental nutrients, and nanomaterials can reduce Cd toxicity and accumulation in plants (Ali et al., 2013a; Ahmad et al., 2016; Wang et al., 2017). The application of these compounds may be an effective way of reducing Cd uptake by plants.

Nanomaterials are a new class of materials that are being increasingly used in agriculture. NPs are available in sizes ranging from 1–100 nm, and they possess some unique properties owing to their small size (Cele, 2020; Seleiman et al., 2021). Recent research on numerous plant species has highlighted the role of nanotechnology methods in agriculture, including tolerance to abiotic pressures such as heavy metals (Konate et al., 2017; Paramo et al., 2020). Particularly, the application of ZnONPs has attracted considerable interest, which can provide up to 80% of the zinc (Zn) in green leafy vegetables. Certain studies suggest that ZnONPs may have positive effects on plants by promoting plant growth and increasing nutrient accumulation (Rawashdeh et al., 2020; Seleiman et al., 2020a; Faizan et al., 2021a). In addition, ZnONPs reduce oxidative damage induced by salt stress (Ali et al., 2022a). Owing to their large surface area and high adsorption capacity, they can trap free ions of heavy metals like Cd (Khan et al., 2017; Rossi et al., 2018). ZnONPs reduce the negative effects of Cd exposure on plants by enhancing growth and antioxidant capacity, increasing chlorophyll content, reducing ROS compounds, and reducing leaf electrolyte leakage (Bashir et al., 2021; Ali et al., 2022b; Chen et al., 2022). They also reduce the Cd content in plants (Ali et al., 2019; Adrees et al., 2021). However, high concentrations of ZnONPs have been found to enhance the bioavailability of Cd, thus leading to elevated concentrations of Cd in rice, which was deleterious to plant growth (Zhang et al., 2019). However, past studies have focused on food crop foliar sprays to address micronutrient deficiencies in crops (Hussain et al., 2018; Rizwan et al., 2019b). Little is known about the potential of ZnONPs for growth, metal accumulation and resistance to abiotic stresses in green leafy vegetables, particularly low concentrations of Cd stress. Therefore, it is important to investigate the effect of ZnONPs on green leafy vegetables under low concentration of Cd stress. Hydroponics is the current method of choice for growing green vegetables. In addition to the possibility of contamination from hydroponic irrigation systems, leafy greens are particularly vulnerable to urban atmospheric deposition due to their huge leaf area (Sharma et al., 2008). This is concerning because leafy green vegetables are often consumed directly without further processing. Lettuce (*Lactuca sativa* L.) is a common vegetable that is widely cultivated worldwide (Meng et al., 2009). It is rich in various vitamins, dietary fiber, and various nutritional minerals, with high nutritional value and benefits to human health (Meng et al., 2009). It also has a high ability to absorb Cd from the soil and does not show signs of Cd toxicity, which adversely affect human health (Cobb et al., 2000). This necessitates the identification of methods for reducing Cd uptake and accumulation in lettuce.

In this study, we investigated the effects of ZnONPs on Cd-stressed lettuce using hydroponics and attempted to understand the putative mitigation mechanisms. This study aimed to determine (a) whether the addition of ZnONPs to the nutrient solution could reduce Cd toxicity in lettuce, (b) the optimal concentration of ZnONPs for reducing Cd toxicity, and (c) how ZnONPs might protect lettuce from Cd toxicity.

## Materials and methods

### Plant material and growing conditions

The experiment was conducted at the College of Horticulture, Gansu Agricultural University, Lanzhou, Gansu Province, PR China (36°03′N, 103°40′E). The ‘Lvsu’ lettuce variety, a Cd-sensitive genotype according to previous studies, was used for this experiment (Dawuda et al., 2019). In an artificial climate chamber at 75%–85% humidity, 24°C day and night, and 250 umol m^-2^·s^-1^ light intensity. Germination was carried out using 50-hole cavity trays filled with vermiculite, and a total of 400 seeds were sown: two seeds were sown in each hole. When lettuce seedlings reached the fifth day of growth, four plants of similar size and growth shape were selected and transplanted into hydroponic containers (length 15 cm; width 10 cm; height 10 cm) containing 1.2 l of 1/4 Hoagland nutrient solution. For the first 4 days, a 1/4 strength Hoagland nutrient solution (pH = 6.0) was administered. Afterwards, a half-strength Hoagland nutrient solution was used. Every four days, the nutrient solution was changed until the plants were ready to be picked and data was collected.

### Experimental design and treatments

In the first experiment, a 4 × 2 factorial experiment in a completely randomized design was conducted using 7-day-old seedlings in a climate-controlled chamber and replicated three times. A total of seven treatments were set up, there were 30 lettuce seedlings in each treatment, which we describe as (1) control (CK), (2) Cd (with the addition of 1 mg/l of Cd), (3) Cd + 1 NPs (with the addition of 1 mg/l of Cd and 1 mg/l of ZnONPs), (4) Cd + 2.5 NPs (with the addition of 1 mg/l of Cd and 2.5 mg/l of ZnONPs), (5) Cd + 5 NPs (1 mg/l of Cd and 5 mg/l of ZnONPs were added), (6) Cd + 10 NPs (1 mg/l of Cd and 10 mg/l of ZnONPs were added), and (7) Cd + 15 NPs (1 mg/l of Cd and 15 mg/l of ZnONPs were added). ZnONPs were purchased from Bio Yuanye (Shanghai, China) with a purity of 99% and a diameter of 30 ± 10 nm. The Cd concentration was 1 mg/l (CdCl_2_-2.5 H_2_O) andwas selected based on the average Cd content recorded in the farmland (Bi et al., 2018). Subsequently, the optimal dose of ZnONPs for the reduction of Cd toxicity was determined based on lettuce growth, chlorophyll content, root activity, reduced Cd accumulation, and relative electrical conductivity (REC) of leaves.

In the second part of the experiment, we selected the optimal concentration of ZnONPs (2.5 mg/l) and observed its effects on growth, oxidative stress, antioxidant enzymes, phytochelatins, and lignin. According to preliminary experiments, the four treatments were described as follows: T0 represented no addition of Cd and ZnONPs; T1 represented 1 mg/l Cd; T2 represented 1 mg/l Cd + 2.5mg/l ZnONPs, and T3 represented 2.5mg/l ZnONPs. The solutions were refreshed daily. Each treatment consisted of three duplicates, each of which had four plants. After 14 days of treatment, the seedlings were harvested for further examination.

### Determination of cadmium content, cadmium transfer factor, and trace element content in lettuce

The solution of concentrated HNO_3_ and perchloric acid (3:1, v: v) was used to digest the dried leaf and root on a hot plate. Prior to heating, the samples were pre-digested for 24 hours in their natural habitat. A Varian Spectra AA 220/Fs atomic absorption spectrophotometer (Mulgrave, Australia) was used to determine the amount of Cd and trace elements in the digests.

The rate of translocation factor (TF) was calculated according to Melo et al. (Melo et al., 2009). TF = Cd concentrationin in leaf/Cd concentrationin in root

### Measurement of lettuce biomass, relative leaf conductivity, chlorophyll content and root activity

After treatment for 15 days, lettuce seedlings were collected and photographed. After separating the samples into leaves and roots, their fresh weight was measured immediately using an electronic scale. The samples were dried in an oven at -80°C until they reached a constant weight, and then their dry weight was determined. Leaf REC was measured using the method reported by Zhou (Zhou et al., 2013). Chlorophyll content was measured by 80% acetone extraction after 14 days of lettuce growth (Tang et al., 2021); root activity was analyzed using the triphenyltetrazolium chloride (TTC) method (Zhang et al., 2013).

### Determination of malondialdehyde, hydrogen peroxide and superoxide radicals

The levels of lipid peroxidation in leaves and roots were estimated using the MDA content. MDA concentrations in lettuce tissue were determined using the method reported by Li et al. (Li et al., 2013) with modifications. The content of H_2_O_2_ and O_2_^-^ in lettuce tissue was determined using a modified method reported by He et al. (He et al., 2011).

### Histochemical detection of hydrogen peroxide and superoxide radicals

The method reported by Tang et al. (Tang et al., 2021) was slightly modified then used for the visualization of H_2_O_2_ and O_2_^-^ levels in the leaves. The H_2_O_2_ accumulation in leaves was detected viay the 3-diaminobenzidine (DAB) staining method by incubating leaves with a DAB solution containing 1 mg/mL (pH = 3.8) and then filtering for 60 minutes using a vacuum extractor. The leaves were then left in the dark for 24 hours. The accumulation of O_2_^-^ in the leaves was observed by staining with nitro blue tetrazolium (NBT). Leaves were removed from the plants and immediately immersed in a solution containing 1 mg/l of NBT (pH = 7.8), vacuum filtered for 20 min, and subsequently incubated for 2 hours at 25°C in the dark.

### Determination of assays for antioxidant enzymes

The extraction solution was 100 mM potassium phosphate buffer (pH 7.0) with 1 mM EDTA-2Na and 1% polyvinylpyrrolidone (PVP). The homogenate was centrifuged at 12,000 g for 20 minutes, and the supernatant was used for the enzyme assay that followed. All operations were carried out at 4°C.

SOD activity was determined using the modified nitro-blue tetrazolium (NBT) method (Wang et al., 2021). First, 0.1 mL of the extract was added to a 50 mM potassium phosphate buffer (pH 7.8) test combination containing 750 mM NBT, 130 mM methionine, 20 mM riboflavin, and 100 mM EDTA. For 20 minutes, the reaction system was illuminated at a light intensity of 4000 lux. Using a spectrophotometer, the amount of enzyme needed to stop 50% of the photoreduction of NBT at 560 nm was set as one unit of SOD.

The POD activity was determined using the modified guaiacol method reported by Iannone et al. (Iannone et al., 2021). The enzyme extract was added to a reaction mixture containing 50 mM potassium phosphate buffer (pH 7.8), 25 mmol/L guaiacol, and 200 mmol/L H_2_O_2_. A spectrophotometer was used to look at the change in absorbance at 470 nm caused by the oxidation of guaiacol.

The determination of CAT activity was performed in the manner described by Tang et al., with modifications. First 0.2 ml of 200 mM H_2_O_2_ was added to a tube containing 50 mM Tris-HCl buffer (pH =7.0) and 0.1 ml of enzyme extract. Then watch the reaction mixture’s absorbance change at 240 nm was observed for 1 minute using a spectrophotometer.

### Determination of phenylalanine, proline, and phenylalanine ammonia lyase activity in lettuce

Lettuce sample preparation and LC-MS analysis of phenylalanine (Phe) and proline (Pro) components were carried out according to the method described by Jin et al. (Jin et al., 2021). The activity of the PAL was determined using the method described by Patel et al. (Patel et al., 2020) with modifications.

### Determination of lettuce total thiol, glutathione, and phytochelatin

The total thiol of the plants was determined using the method described by Krippner and Schubert et al. (Krippner and Schubert, 2021), with minor changes. The contents of GSH were determined using the method described by Jinadasa et al. (Jinadasa et al., 2016). The PCs were measured using the following formula: PCs = total thiol – GSH (Bhargava et al., 2005).

### Histochemical detection of lignin and determination of peroxidase activity levels in roots and determination of lignin contents

A phloroglucinol-HCl solution was used to determine the lignin content of roots (Fukushima and Hatfield, 2004). Roots were incubated in a solution of 1% (w/v) phloroglucinol generated in 6N HCl for 5 minutes before being washed with distilled water and placed on glass slides. Pyrogallol was utilized to determine the peroxidase activity of the cell wall (cwPODs) (Rogers et al., 2005). For 15 minutes, the lettuce root tips were immersed in this solution, cleaned thoroughly, and mounted on glass slides.

As for lignin, lettuce roots were extracted to obtain protein-free cell wall fractions (Finger-Teixeira et al., 2010). Then, the bromoacetylation method (Eleftheriou et al., 2015) was used to measure the amount of lignin in the roots (Eleftheriou et al., 2015).

### Statistical analysis

The experiment was repeated three times in a completely randomized fashion. The dataset was normalized using IBM SPSS Statistic 20 software before being analyzed using the analysis of variance (ANOVA) test. Differences were considered to be statistically significant according to Duncan’s test at *P* < 0.05.

## Results

### Effects of zinc oxide nanoparticles on biomass, chlorophyll content, leaf relative electrical conductivity and root activity of lettuce under cadmium stress

**Figure 1:**
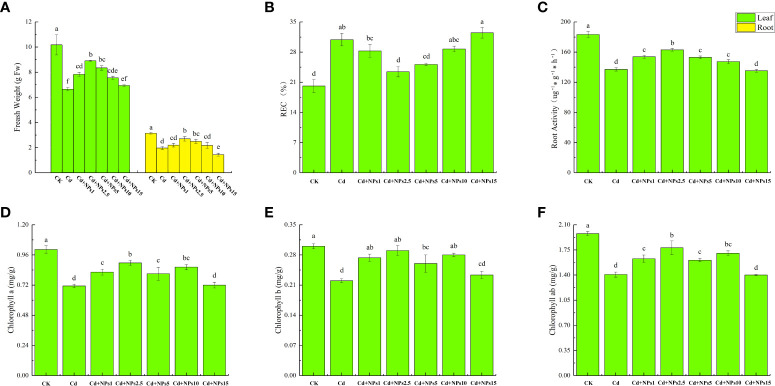
Effects of different concentrations of zinc oxide nanoparticles (ZnONPs) on biomass accumulation **(A)**, leaf relative electrical conductivity (REC) **(B)**, root activity **(C)** and chlorophyll content **(D–F)** in lettuce under cadmium (Cd) stress. CK, no treatment; Cd, exposed to 1 mg/l of Cd; Cd + 1 Nps, exposed to 1 mg/l of Cd and 1 mg/l of ZnONPs; Cd + 2.5 Nps, exposed to 1 mg/l of Cd and 2.5 mg/l of ZnONPs; Cd + 5 Nps, exposed to 1 mg/l of Cd and 5 mg/l of ZnONPs; Cd + 10 Nps, exposed to 1 mg/l of Cd and 10 mg/l of ZnONPs; Cd + 15 Nps, exposed to 1 mg/l of Cd and 15 mg/l of ZnONPs. Data are shown as means ± SE of three replicates. Bars marked by different letters indicate significant differences according to Duncan’s test (p < 0.05).

As shown in **Figure 1**, Cd stress decreased biomass, chlorophyll content, and root activity of lettuce seedlings and increased electrolyte leakage from leaves compared with the control. In experimental concentrations, the effect of ZnONPs at 2.5 mg/l was the most significant. It increased the growth of leaves (34%) and roots (37%), chlorophyll a content (26%), chlorophyll b content (30%), chlorophyll ab content (26%), and root activity (19%); but reduced electrolyte leakage (24%). In addition, the fresh weight of lettuce seedlings, decreased root activity, and increased REC were determined following the addition of 10 mg/l and 15 mg/l of ZnONPs.

### Effect of zinc oxide nanoparticles on trace elemental uptake of lettuce under cadmium stress

**Figure 2:**
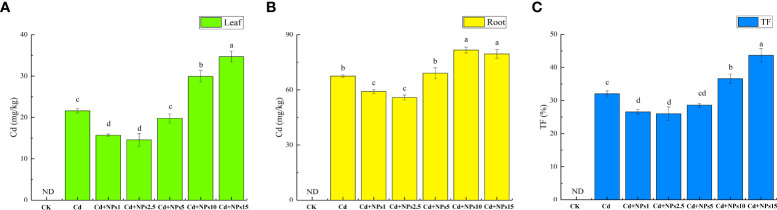
Effect of ZnONPs on Cd content in lettuce leaves **(A)**, Cd content in lettuce roots **(B)**, and Cd translocation factor (TF) under Cd stress **(C)**. T0, no treatment; T1, exposure to 1 mg/l Cd; T2, exposure to 1 mg/l Cd and 2.5 mg/l ZnONPs; T3, exposure to 2.5 mg/l ZnONPs. Bars marked by different letters indicate significant differences according to Duncan’s test (*p* < 0.05).

The effects of ZnONPs on Cd content and translocation factor in lettuce seedlings are shown in **Figure 2**. The addition of 1 mg/l and 2.5 mg/l of ZnONPs significantly reduced the Cd content and translocation factor in lettuce seedlings, and decreased the Cd content in leaves and roots by 27%/12% and 33%/17%, respectively. The addition of 5 mg/l ZnONPs reduced the Cd content in leaves (8%), but had no significant effect on the Cd content in roots and the translocation factor. However, the addition of 10 mg/l and 15 mg/l of ZnONPs increased the Cd content in leaves and roots. Application of 1 mg/l, 2.5 mg/l, and 5 mg/l ZnONPs reduced the Cd translocation factor, with 1 mg/l and 2.5 mg/l ZnONPs, thus reducing the Cd translocation factor considerably.

**Table 1** shows the effect of ZnONP application on the uptake of elemental nutrients by lettuce exposed to Cd. Cd stress resulted in a significant decrease in the uptake of copper (Cu), iron (Fe), manganese (Mn), and zinc (Zn) by lettuce leaves (10%, 42%, 30% and 34%, respectively), and an increase in the uptake of calcium (Ca) (3%), however and no significant effect on the uptake of magnesium (Mg). The accumulation levels of Fe, Mn, Cu and Zn in the roots decreased by 10%, 28%, 44%, and 11%, respectively, while the accumulation levels of Mg and Ca in the roots did not change significantly.The effects of ZnONPs application on different elemental nutrients in lettuce leaves varied, with the uptake of Cu, Fe, and Mn by leaves initially increasing and then decreasing. The uptake of Zn, Mg, and Ca was positively correlated with the concentration of ZnONPs. The application of ZnONPs also promoted nutrient uptake in the roots, where the accumulation of Zn and Ca was positively correlated with the concentration of ZnONPs. The accumulation of Cu, Fe, and Mn increased under the treatment of low and moderate concentrations of ZnONPs but decreased under the treatment of high concentrations of ZnONPs.

**Table 1:** Effects of ZnONPs on nutrient elements uptake by lettuce under Cd stress.

Treatment	Elements content (mg kg^−1^ DW)						
	Cu		Fe	Mg	Mn	Zn	Ca
**Leaves**							
CK	13.03 ± 0.05a		185.49 ± 2.03a	3952.29 ± 10.35e	131.88 ± 0.26c	39.36 ± 0.25f	3978.38 ± 23.93f
Cd	11.79 ± 0.22b		107.02 ± 0.24d	3991.2 ± 29.52e	123.45 ± 0.31d	25.87 ± 0.95g	4098.67 ± 26.15e
Cd + 1 Nps	12.06 ± 0.22b		119.2 ± 2.26c	4156.69 ± 17.35d	130.7 ± 2.95c	88.64 ± 6.58e	4241.97 ± 46.15d
Cd + 2.5 Nps	13.15 ± 0.21a		126.64 ± 0.6b	4272.27 ± 43.64c	144.5 ± 1.49a	199.33 ± 3.79d	4396.1 ± 24.52c
Cd + 5 Nps	13.03 ± 0.21a		117.78 ± 3.01c	4365.61 ± 29.31b	147.7 ± 0.76a	245.99 ± 7.22c	4498.79 ± 25.36ab
Cd + 10 Nps	12.64 ± 0.15a		107.78 ± 3.01d	4493.81 ± 24.55a	139.05 ± 0.81b	357.06 ± 6.56b	4452.15 ± 22.62bc
Cd + 15 Nps	12.07 ± 0.18b		85.39 ± 1.22e	4529.96 ± 33.45a	123.55 ± 1.28d	657.08 ± 12.23a	4554.54 ± 32.1a
**Roots**							
CK	19.4 ± 0.19a		3014.75 ± 49.52a	1015.65 ± 11.86bc	99.03 ± 1.37a	131.84 ± 1.24f	1031.53 ± 26.02c
Cd	17.37 ± 0.13d		2154.63 ± 18.34e	950.68 ± 8.04c	55.72 ± 1.21de	117.19 ± 0.56g	1053.32 ± 33.34c
Cd + 1 Nps	17.97 ± 0.16bc		2324.65 ± 32.93cd	1049.19 ± 23.62b	63.51 ± 1.27c	184.73 ± 0.44e	1191.78 ± 18.8b
Cd + 2.5 Nps	18.49 ± 0.32b		2480.77 ± 88.78b	1183.18 ± 35.59a	69.36 ± 2.08b	338.78 ± 2.17d	1215.71 ± 11.35b
Cd + 5 Nps	18.66 ± 0.33b		2402.69 ± 30.78bc	1155.78 ± 10.15a	58.99 ± 1.36d	389.4 ± 2.87c	1200.2 ± 20.5b
Cd + 10 Nps	18.06 ± 0.26bc		2256.75 ± 18.24de	1028.98 ± 29.93b	57.07 ± 1.32de	710.28 ± 3.76b	1255.61 ± 31.64ab
Cd + 15 Nps	17.53 ± 0.22d		2161.33 ± 17.24e	955.43 ± 20.68c	53.36 ± 0.94e	1274.09 ± 5.54a	1311.92 ± 40.51a

### Effect of zinc oxide nanoparticles on oxidative stress of lettuce under cadmium stress

**Figure 3:**
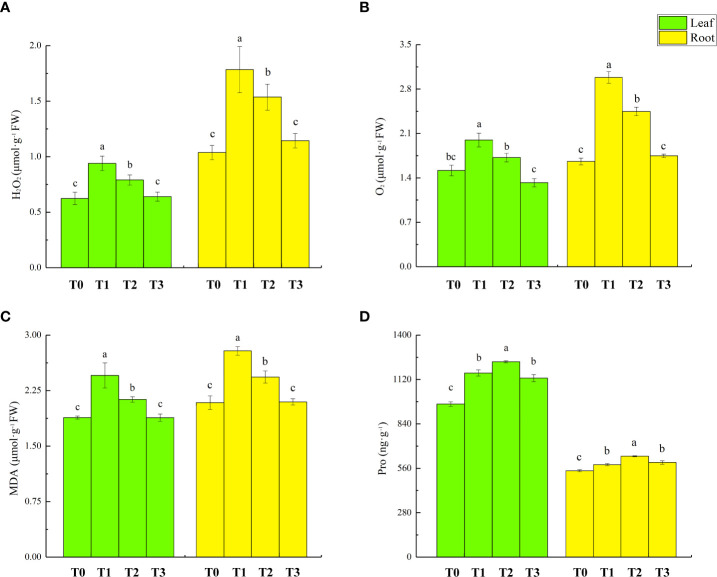
Effect of ZnONPs on hydrogen peroxide (H_2_O_2_) content **(A)**, superoxide radicals (O_2_^-^) content **(B)**, malondialdehyde (MDA) content **(C)** and proline (Pro) content **(D)**, in leaves and roots of lettuce. T0, no treatment; T1, exposure to 1 mg/l Cd; T2, exposure to 1 mg/l Cd and 2.5 mg/l ZnONPs; T3, exposure to 2.5 mg/l ZnONPs. Data are shown as means ± SE of three replicates. Bars marked by different letters indicate significant differences according to Duncan’s test (*p* < 0.05).

The overproduction of reactive oxygen species is one of the main adverse effects of Cd stress on plants. The accumulation of ROS in lettuce seedlings with different treatments is shown in **Figure 3**. The levels of H_2_O_2_ in the shoots and roots of T1 treatment lettuce seedlings increased by 50% and 72%, respectively. Furthermore, the levels of O_2_ in shoots and roots increased by 45% and 59%, respectively. In T2 treatment, H_2_O_2_ and O_2_ content of lettuce seedlings decreased by 16% and 14%, respectively, in lettuce seedling leaves and by 14% and 14% in root, respectively.

The accumulation of malondialdehyde (MDA) content in lettuce seedlings with different treatments is shown in **Figure 3C**. Compared to T0 treatment, the MDA levels in lettuce seedling’s leaves and roots of T1 treatment increased by 30% and 34%, respectively. There was no significant change in the Pro of lettuce seedling tissues in T3 treatment compared to T1 treatment. The accumulation of malondialdehyde (MDA) content in lettuce seedlings following different treatments is shown in **Figure 3D**. Compared with T0 treatment, the Pro levels in the leaves and roots under T1 treatment’s lettuce seedlings increased by 20% and 7%, respectively; T2 treatment further increased the Pro content in leaves and roots by 6% and 9%, respectively, from the levels in T1 treatment. Compared to T0 treatment, T3 treatment increased the leaf and root Pro by 17% and 10%, respectively.

**Figure 4:**
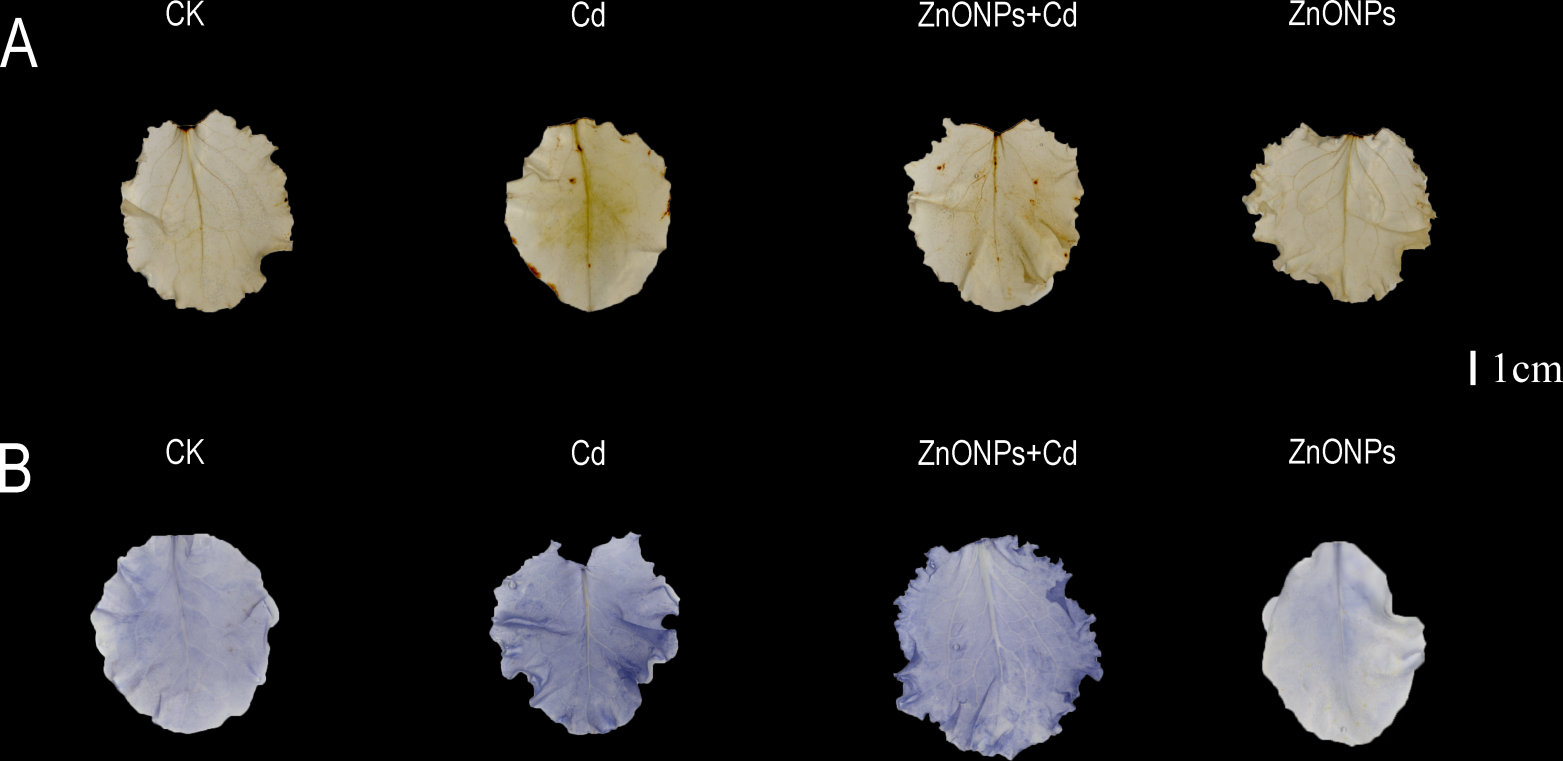
H_2_O_2_ **(A)** and O_2_^-^ **(B)** detection of leaf diaminobenzidine and nitro blue tetrazolium staining. Bar= 1 cm. Brown and blue colors indicate the amount of H2O2 and O2- in lettuce leaves, and small amounts of brown and blue colors indicate less accumulation of H_2_O_2_ and O_2_^-^ in the leaves.

The leaf H_2_O_2_ and O_2_^-^ detection of DAB and NBT staining are shown in **Figure 4**. In DAB staining, there was less brown color in the leaves in T2 treatment than those in T1 treatment. In contrast, T3 treatment showed no significant color change compared to T0 treatment. In NBT staining however, T2 treatment had less blue color compared to T1 treatment. There was no significant color change in T3 treatment compared to T0 treatment.

### Effect of zinc oxide nanoparticles on antioxidant enzymes of lettuce under cadmium stress

**Figure 5:**
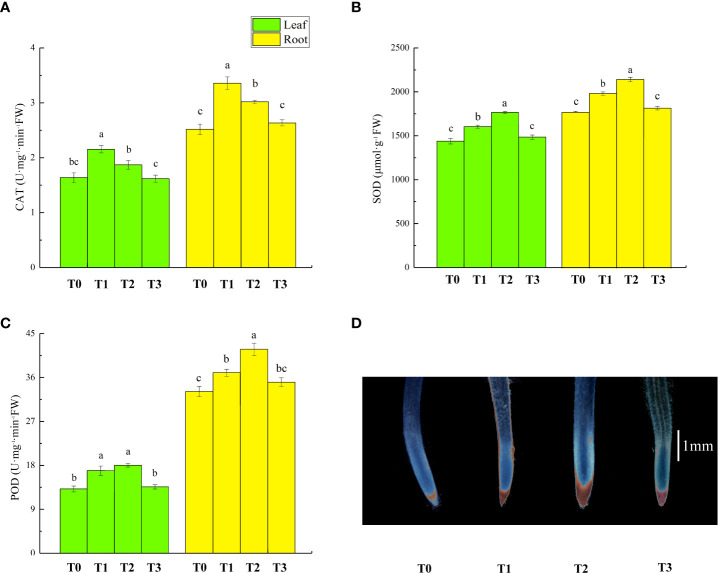
Effects of ZnONPs on the activities of catalase (CAT, **A**), superoxide dismutase (SOD, **B**), and peroxidase (POD, **C**) in lettuce seedlings and pyrogallol staining of lettuce root tips **(D)**. T0, no treatment; T1, exposure to 1 mg/l Cd; T2, exposure to 1 mg/l Cd and 2.5 mg/l ZnONPs; T3, exposure to 2.5 mg/l ZnONPs. Brown color indicates the activity of cwPOD in lettuce roots and a large amount of brown color indicates higher cwPOD activity in roots, bar= 1 mm. Data are shown as means ± SE of three replicates. Bars marked by different letters indicate significant differences according to Duncan’s test (*p* < 0.05).

The activities of antioxidant enzyme in lettuce seedlings following different treatments are shown in **Figure 5**. Compared to T0 treatment, the activities of catalase (CAT), superoxide dismutase (SOD) and peroxidase (POD) in leaves and roots of plants that received T1 treatment increased by 32%/33%, 12%/12% and 29%/15%, respectively. Compared to T1 treatment, T2 treatment decreased CAT activity in lettuce leaves and roots by 13% and 10%, and increased SOD and POD activity in leaves and roots by 10%/8% and 13% in root. However, there was no significant change in leaf POD activity. T3 treatment had no significant effect on the antioxidant enzymes’ activity compared to T0 treatment. The histochemical staining of root cwPOD activity is shown in **Figure 5D**, with more brown indicating relatively strong cwPOD activity. T1 and T3 treatment resulted in more brown color than T0 treatment. The brown fraction was relatively more in T2 treatment, indicating the highest cwPOD activity.

### Effect of zinc oxide nanoparticles on phenylalanine content, phenylalanine ammonia lyase activity, and lignin content of lettuce under Cd cadmium

**Figure 6:**
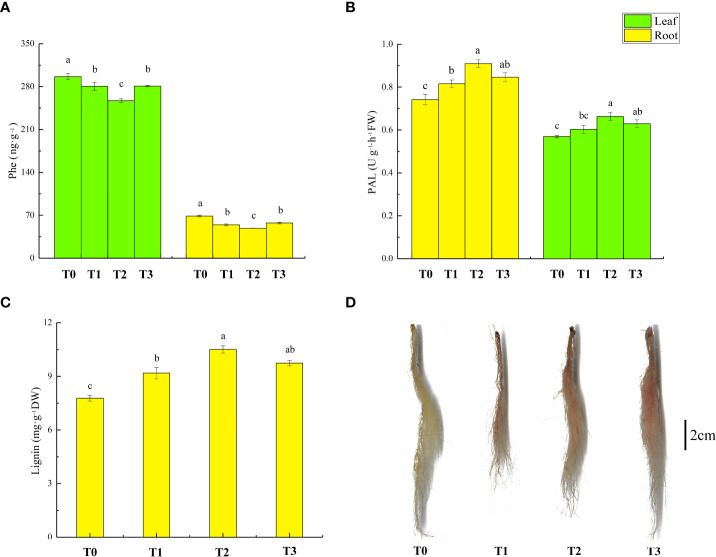
Effect of ZnONPs on Phe content **(A)** and PAL activity **(B)** in leaves and roots of lettuce, and lignin content **(C)** roots of lettuce and visualization of lignin staining in lettuce roots **(D)**. T0, no treatment; T1, exposure to 1 mg/l Cd; T2, exposure to 1 mg/l Cd and 2.5 mg/l ZnONPs; T3, exposure to 2.5 mg/l ZnONPs. The small amount of reddish-brown color indicates that the lignin accumulation in the roots is low, bar = 2cm. Data are shown as means ± SE of three replicates. Bars marked by different letters indicate significant differences according to Duncan’s test (*p* < 0.05).

The Phe content, PAL activity in lettuce sedding’s leaves and roots, and Lignin content in roots are shown in **Figure 6**. The Phe content in leaves and roots decreased by 5% and 21% in T1 treatment compared with T0 treatment; PAL activity increased by 10% in roots and did not change significantly in leaves; and lignin content in roots increased by 18%. T2 treatment further reduced Phe content in lettuce leaves and roots (8% and 10%) compared to T1 treatment, increased PAL activity (10% and 11%), and increased root lignin content (14%). Compared to T0 treatment, T3 treatment decreased the Phe content in leaves and roots (5% and 17%) and increased PAL activity (11% and 14%) and lignin content (6%).

The staining of the root lignin is shown in **Figure 6D**, with more red color representing higher lignin content. T1 and T3 treatment increased the red color of the roots. The red color was more in T2 treatment than in T1 treatment.

### Effect of zinc oxide nanoparticles on the content of total thiol, glutathione and phytochelatins in different parts of lettuce under cadmium stress

**Figure 7:**
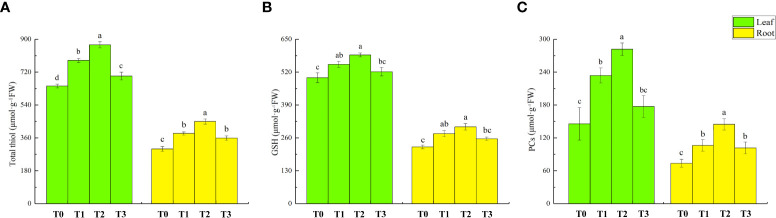
Effect of ZnONPs on total thiol content **(A)**, GSH content **(B)** and PCs content **(C)** and proline (Pro) content in leaves and roots of lettuce. T0, no treatment; T1, exposure to 1 mg/l Cd; T2, exposure to 1 mg/l Cd and 2.5 mg/l ZnONPs; T3, exposure to 2.5 mg/l ZnONPs. Data are shown as means ± SE of three replicates. Bars marked by different letters indicate significant differences according to Duncan’s test (p < 0.05).

**Figure 7** shows the contents of total thiol, GSH, and PCs in the leaves and roots of lettuce seedlings under different treatments. Compared to T0 treatment, the content of total thiol, GSH and PCs in the leaves under T1 treatment increased by 22%, 11%, and 61%, respectively. The amount of total thiol, GSH, and PCs in the roots increased by 28%, 23%, and 44%, respectively. The content of total thiol and PCs in the leaves further increased by 11% and 20.5% following T2 treatment compared to T1 treatment. In the root, the amount of total thiol and PCs increased by 17% and 36%, respectively. However, the GSH content did not increase significantly. Compared to T0 treatment, T3 treatment increased the total thiol content of lettuce leaves and roots (9% and 20%) and the PC content of roots (38%). There was no significant effect on the GSH content of leaves and roots.

## Discussion

Recent research has confirmed the role of nanomaterials in enhancing plant tolerance to abiotic stresses like metals (Faizan et al., 2021b; Ali et al., 2022a). ZnONPs are widely used nanomaterials. They release Zn, an essential micronutrient for regulating plant development and exhibits lowtoxicity compared to other forms of Zn (Kouhi et al., 2014; Khoshgoftarmanesh and Markarian, 2022). Many studies have reported the growth-promoting effects of ZnONPs in a variety of plants. Song and kimfound that it increased the biomass of lettuce and carrots (Song and Kim, 2020).Venkatachalam et al. (Venkatachalam et al., 2017) found that it increased the photosynthetic pigment content of the leaves of silver acacia seedlings. The negative effects of Cd on plants have been described in the introduction section. Owing to its resemblance to phosphorus, Cd is easily transported by plants and accumulates in edible parts, whichnegatively impacts plant growth (Huang et al., 2020). In addition, Cd stress causes imbalances of trace elements (Wang et al., 2019). As a result, this study aims to elucidate potential mechanisms of ZnONPs regulation of Cd tolerance in lettuce. The study results show that Cd stress significantly reduced the biomass, chlorophyll, and root activity of lettuce seedlings, and significantly increased REC (**Figure 1**). Cd causes ultrastructural changes and induces programmed cell death, which is one of the main factors contributing to decreased chlorophyll content, root activity and increased leaf REC (Ali et al., 2015; Rizwan et al., 2016). Moreover, Cd competes with other trace elements for binding sites, thus reducing the uptake of trace elements (Rizwan et al., 2017; Ronzan et al., 2018). In this study, the supply of ZnONPs alleviated the toxic effects of Cd stress on lettuce seedlings, reduced Cd uptake and translocation, and increased the trace elements in lettuce. Similar findings have been recorded in different studies onplants (Rizwan et al., 2019b; Faizan et al., 2020). Zn released from ZnONPs maintains the stability of biological cell membranes, reduces electrolyte leakage and loss of cell viability caused by Cd stress, and positively regulates the expression of metal transport proteins in plants (Mustafa and Komatsu, 2016; Rajapakse et al., 2017; Hussain et al., 2018). Furthermore, appropriate concentrations of Zn can reduce the transfer of Cd from roots to shoots by altering the expression pattern of *ZIP* genes (Palusinska et al., 2020). This may be the reason underlying the alleviation of Cd stress in lettuce seedlings by ZnONPs. However, in this experiment, high concentrations of ZnONPs were found to be toxic to lettuce seedling growth and to increase Cd content and transport. This could be the result of toxic effects caused by a large accumulation of Zn, with potential phytotoxicity occurring when the Zn concentration in plant leaf tissue exceeds 200 mg/kg dry matter. High concentrations of ZnONPs have been observed in previous studies to lead to a significant accumulation of Zn in plants, leading to toxicity (Bonnet et al., 2000; Srivastav et al., 2021; Hong et al., 2022). High concentrations of Zn can also have indirect effects on the uptake and mutual elemental interactions of other elements, thus increasing the accumulation of Cd (Zhang et al., 2019; Li et al., 2021; Xiao et al., 2022). Therefore, attention should be paid to the concentration of ZnONPs to avoid their toxic effects on plants. Notably, low and moderate concentrations of ZnONPs have a significant effect on Fe uptake, and improve Fe accumulation in leaves more than in roots, which is consistent with the findings of Sharifan et al. (Sharifan et al., 2019). Because ZnONPs at a concentration of 2.5 mg/l alleviated the toxicity of Cd to lettuce seedlings and minimized the accumulation of Cd, were selected for subsequent experiments to investigate the possible mechanism of Cd stress alleviation by ZnONPs.

The most common effect of Cd toxicity is the induction of ROS production and accumulation, which can cause oxidative damage to plants, leading to biofilm damage and promoting increased MDA content owing to lipid peroxidation (Noman et al., 2018a). Generally, higher plants scavenge excess ROS by increasing antioxidant enzyme activity to overcome ROS accumulation and membrane lipid peroxidation caused by Cd toxicity. When plants experience an oxidative burst, SOD first converts O_2_ to the less toxic H_2_O_2_, after which CAT and POD are responsible for scavenging H_2_O_2_ (Noman et al., 2018b). The H_2_O_2_, O_2_ and MDA content of lettuce seedlings were significantly increased under Cd stress, and significant enhancement of SOD, POD, and CAT activities were observed. Previous studies have obtained similar results for other plants (Xu et al., 2015; Zouari et al., 2016).The use of ZnONPs reduced H_2_O_2_ content, O_2_ and MDA content and enhanced SOD and POD activities in lettuce seedlings. Numerous studies have documented ZnONP-mediated induction of antioxidant enzyme activities in different plants. Rizwan et al. found that ZnONPs increased the activity of antioxidant enzymes and reduced the accumulation of reactive oxygen species in cowpea seedlings (Rizwan et al., 2019a). Pavani et al. found that ZnONPs increased SOD and POD activities in response to different types of abiotic stress (Pavani et al., 2020). However, CAT activity decreased after the application of ZnONPs, which could be due to the decrease in H_2_O_2_ content. Mitigation of Cd stress toward ZnONPs was mainly achieved by enhancing SOD and POD activities and decreasing ROS content. The sole application of ZnONPs had no significant effect on ROS content and antioxidant enzyme activity in lettuce seedlings, indicating that the concentrations used were appropriate and did not cause Zn toxicity.

To withstand metal stress, plants accumulate a variety of metabolites. Pro is an important amino acid metabolite that plays a crucial role in plant tolerance to stress conditions and can act as a plant osmoregulatory substance, metal chelator, and ROS quencher (Ghasemi-Omran et al., 2021). It can stabilize and protect the functions of various enzymes, regulate plant growth, and play an important role in mitigating heavy metal toxicity (Torabian et al., 2016; Hussain et al., 2021). In this study, both Cd stress and ZnONPs increased the amount of proline in lettuce. The highest levels of proline were found in lettuce seedlings with ZnONPs applied under Cd stress, and similar results were found in a previous study (Faizan et al., 2021b). In addition, ZnONPs could potentially increase proline content, and previous studies have found that the supply of ZnONPs in previous reports increases Pro content in plants (Faizan et al., 2020), which may be related to the increased expression of proline biosynthesis genes (Faizan et al., 2021a). Generally, there are two pathways of Cd transport to xylem: the symplastic pathway and the apoplast pathway (Dong et al., 2017; Qi et al., 2020). The transport of Cd through the apoplast pathway mainly involves the transmembrane transport of thiol compounds and ions (Silveira Rabelo et al., 2017; Song et al., 2017). Plants minimize the toxicity of Cd using thiols, glutathione (GSH), and cysteine-rich metal-binding peptides (PCs) in root hairs to bind to Cd and immobilize it in vesicles thus limiting its transportation (Yan et al., 2020). This study documented a significant increase in thiol, GSH, and PCs content in lettuce seedlings under Cd stress and ZnONPs alone. In addition, the supply of ZnONPs further increased thiol, GSH, and PCs content in the tissues of lettuce seedlings under Cd stress. This suggests that ZnONPs could promote the synthesis of thiol compounds as a way of reducing the toxic effects of Cd. This may be the effect of Zn released from ZnONPs. Zn induces the synthesis of sulfur-containing compounds including GSH and PCs, thereby increasing the levels of plant PCs and GSH (Sharifan et al., 2020). Similar results have been reported in previous studies. Wei et al. found that Zn promotes the accumulation of sulfate in plants and increases the content of cysteine, which in turn increases the content of thiols and PCs (Wei et al., 2022); Howladar et al. found that ZnONPs promote the ASA-GSH cycle and increase GSH content in tissues (Howladar, 2022; Prakash et al., 2022). Therefore, ZnONPs increased the accumulation of thiols in lettuce seedlings, which is significant to improving their tolerance to Cd toxicity.

In the symplastic pathway Cd was absorbed by the root system; it was first transported to the xylem and then to the stem and leaves (Tao and Lu, 2022). PAL plays an important role because it catalyzes the conversion of phenylalanine to lignin, thereby increasing the level of lignification. The increased level of lignification enhances the plastid barrier, reducing cell wall penetration and preventing Cd entry into the cytoplasm. In addition, lignin in the root cell wall provides Cd binding sites that can protect protoplasts from Cd stress by anchoring Cd in the cell wall, which is an effective way of preventing Cd transport into the leaves (Kidwai et al., 2019). This study found that both Cd stress and ZnONPsincreased the lignin content in the roots of lettuce seedlings. Moreover, the highest lignin content was found in the roots of lettuce seedlings with ZnONPs applied under Cd stress, which is consistent with the results of previous experiments. Similar findings have been found in previous experiments. Zhao et al. found that plants could reduce the toxicity of Cd by increasing lignin to fix Cd (Zhao et al., 2021). Benakova et al. found that low concentrations of ZnONPs were also able to increase the lignin content of roots (Benakova et al., 2017). In contrast, Molnar et al. (Molnar et al., 2020) found maximum lignin content in co-treatments of Zn and Cd in Brassica napus. This could be related to the increased activity of PAL and cwPOD, both key enzymes in the synthesis of plant lignin. Cd stress and ZnONPs also increased PAL activity in the present study. A decrease in Phe content was observed owing to Cd stress and the use of ZnONPs, which could be attributed to the increase in PAL activity. PAL is the rate-limiting enzyme of lignin anabolism and can to catalyze the conversion of Phe to cinnamic acid (Hose et al., 2001; Garza-Alonso et al., 2021). This could explain the decrease in Phe content in lettuce seedlings, as a large amount of Phe is involved in lignin synthesis. However, additional studies at the molecular and biochemical levels are required to lucidate the role of ZnONPs in thiol metabolism.

## Conclusion

Cd stress adversely affects lettuce through the inhibition of growth, ROS production, and mineral element uptake. The ZnONPs in the nutrient solutions played a significant role in boosting growth and lowering Cd toxicity. ZnONPs reduce oxidative stress caused by Cd by enhancing the antioxidant system to scavenge ROS production in Cd-exposed plants. ZnONPs also reduce the toxicity of Cd by speeding up thiol metabolism and creating more lignin, which inhibits the movement of Cd from roots to leaves. Additionally, ZnONPs can regulate the uptake and transport of trace elements. This effect is most pronounced in the photosynthetic-related elements, such as Fe, Mg, and Mn. The ability of ZnONPs to attenuate Cd toxicity in lettuce may be achieved by removing excess ROS, inhibiting Cd uptake, and immobilizing it in the roots to reduce its translocation to the leaves. However, more studies at the molecular level are required to fully explain the involvement of ZnONPs in the Cd stress response of lettuce. In conclusion, the application of lower concentrations of ZnONPs to lettuce under Cd stress is an effective detoxification method that can be investigated in phytoremediation.

## Data availability statement

The original contributions presented in the study are included in the article/supplementary material. Further inquiries can be directed to the corresponding author.

## Author contributions

FG and conceived and designed the experiments. analyzed the data. wrote the manuscript. JX conceived, and designed the experiments. JZ contributed reagents/materials/analysis tools. JL contributed towards, execution of different experiments, analyzed the data. XZ experimental design, data processing, related discussions and manuscripts writing improvement. CW contributed towards, execution of different experiments. TN contributed towards execution of different experiments. CT contributed towards, execution of different experiments. All authors contributed to the article and approved the submitted version. 

## Funding

This research was supported by the National Key Research and Development Program (2016YFD0201005), the Gansu Province Special Fund Project for Guiding Science and Technology Development (2018ZX-02), and the Research and Industrialization Demonstration of Greenhouse Structure and Construction Technology for Horticultural Crop Cultivation in Northwest Non-Arable Land (201203002).

## Acknowledgments

I thank my supervisor and colleagues for their help in my experiments. I also thank the laboratory for providing me with the experimental conditions.

## Conflict of interest

The authors declare that the research was conducted in the absence of any commercial or financial relationships that could be construed as a potential conflict of interest.

## Publisher’s note

All claims expressed in this article are solely those of the authors and do not necessarily represent those of their affiliated organizations, or those of the publisher, the editors and the reviewers. Any product that may be evaluated in this article, or claim that may be made by its manufacturer, is not guaranteed or endorsed by the publisher.
